# A phase I trial to assess the pharmacology of the new oestrogen receptor antagonist fulvestrant on the endometrium in healthy postmenopausal volunteers

**DOI:** 10.1038/sj.bjc.6600644

**Published:** 2002-11-26

**Authors:** S Addo, R A Yates, A Laight

**Affiliations:** LCG Bioscience, Bourn Hall Clinic, Bourn, Cambridge, UK; AstraZeneca Pharmaceuticals, Alderley Park, Macclesfield, Cheshire SK10 4TG, UK

**Keywords:** fulvestrant, oestrogen receptor antagonist, postmenopausal, endometrium, pharmacology

## Abstract

While tamoxifen use is associated with clear benefits in the treatment of hormone-sensitive breast cancer, it also exhibits partial oestrogen agonist activity that is associated with adverse events, including endometrial cancer. Fulvestrant (‘Faslodex’) is a new oestrogen receptor antagonist that downregulates the oestrogen receptor and has no known agonist effect. This single-centre, double-blind, randomised, parallel-group trial was conducted to determine the direct effects of fulvestrant on the female endometrium when given alone and in combination with the oestrogen, ethinyloestradiol. Following a 14-day, pretrial screening period, 30 eligible postmenopausal volunteers were randomised to receive fulvestrant 250 mg, fulvestrant 125 mg or matched placebo administered as a single intramuscular injection. Two weeks postinjection, volunteers received 2-weeks concurrent exposure to ethinyloestradiol 20 μg day^−1^. Endometrial thickness was measured before and after the 14-day screening period with further measurements predose (to confirm a return to baseline) and on days 14, 28 and 42 post-treatment with fulvestrant. Pharmacokinetic and safety assessments were performed throughout the trial. Fulvestrant at a dose of 250 mg significantly (*P*=0.0001) inhibited the oestrogen-stimulated thickening of the endometrium compared with placebo. Neither the 125 mg nor 250 mg doses of fulvestrant demonstrated oestrogenic effects on the endometrium over the initial 14-day assessment period. Fulvestrant was well tolerated and reduced the incidence of ethinyloestradiol-related side effects. At the same dose level that is being evaluated in clinical trials of postmenopausal women with advanced breast cancer, fulvestrant (250 mg) is an antioestrogen with no evidence of agonist activity in the endometrium of healthy postmenopausal women.

*British Journal of Cancer* (2002) **87**, 1354–1359. doi:10.1038/sj.bjc.6600644 www.bjcancer.com

© 2002 Cancer Research UK

## 

Non-steroidal antioestrogens such as tamoxifen (‘Nolvadex’) have revolutionised the treatment of breast cancer over the last 30 years. In particular, tamoxifen has become established as the ‘gold standard’ for the treatment of all stages of breast cancer (Early Breast Cancer Triallists Collaborative Group [EBCTCG], 1992; 1998) and has also been shown to have a role in the prevention of primary and contralateral breast cancer (Fisher *et al*, 1998; reviewed by Chlebowski *et al*, 1999; Narod *et al*, 2000; King *et al*, 2001). Although the oestrogenic activity of long-term tamoxifen therapy helps to maintain bone density, (Love *et al*, 1992) and reduce circulating low-density lipoprotein cholesterol (Love *et al*, 1990; Bilmoria *et al*, 1996), it is also associated with partial oestrogen agonist activity linked with endometrial proliferation and an increased risk of endometrial cancer (Fisher *et al*, 1994; 1998; Bergman *et al*, 2000). Compared to non-tamoxifen treated women, the endometrial cancer associated with long-term tamoxifen use has a worse prognosis, probably due to it exhibiting a less favourable histology and higher stage (Bergman *et al*, 2000). These adverse events have therefore raised concerns about the long-term use of tamoxifen. More recently, drugs that inhibit aromatase, and thereby block the conversion of androgen to oestrogen in postmenopausal women, have become valuable options for the treatment of advanced breast cancer.

In contrast to tamoxifen, fulvestrant (‘Faslodex’) is a new oestrogen receptor (ER) antagonist that downregulates cellular levels of the ER and has no known agonist effects. In preclinical studies, fulvestrant shows no oestrogen-like activity and completely ablates the activity of endogenous oestrogens (Wakeling *et al*, 1991). Moreover, magnetic resonance imaging of the uterus in ovarectomised monkeys also suggests that fulvestrant is an effective antioestrogen (Dukes *et al*, 1992, 1993). The data surrounding the clinical potential of fulvestrant in postmenopausal patients with breast cancer have been encouraging (Defriend *et al*, 1994; Howell *et al*, 1996, 2002; Osborne *et al*, 2002). In this group of patients, fulvestrant inhibits tumour cell proliferation associated with a profound decrease in immunocytochemically detectable ER protein (Defriend *et al*, 1994; Robertson *et al*, 2001). One small phase II trial, involving 19 postmenopausal patients with tamoxifen refractory disease, suggested that fulvestrant might have fewer side effects in terms of menopausal symptoms than tamoxifen, with no negative effects being observed on the liver, brain or genital tract (Howell *et al*, 1996). The pharmacokinetic data associated with these studies in breast cancer patients showed that monthly intramuscular (i.m.) injections of fulvestrant 250 mg maintained detectable plasma levels of drug over a period of 28 days and resulted in a marked reduction in levels of ER protein (Howell *et al*, 1996; Robertson *et al*, 2000, 2001). Fulvestrant was shown to be at least as effective as the aromatase inhibitor anastrozole in terms of time to progression, and secondary endpoints including objective response and clinical benefit, in two randomised phase III trials in postmenopausal women with locally advanced or metastatic breast cancer who had progressed following prior endocrine therapy for either advanced or early breast cancer (Howell *et al*, 2002; Osborne *et al*, 2002). Rates of adverse events (AEs) were similar in both arms of the two trials.

There has been no suggestion to date that fulvestrant produces ovarian or endometrial stimulation in postmenopausal women (Defriend *et al*, 1994; Howell *et al*, 1996) and the lack of agonist activity associated with fulvestrant suggests an improved efficacy and tolerability profile relative to tamoxifen. The present trial was conducted in healthy postmenopausal volunteers to specifically assess the pharmacological effects of fulvestrant on the postmenopausal endometrium.

## VOLUNTEERS AND METHODS

### Volunteers

**Figure 1:**
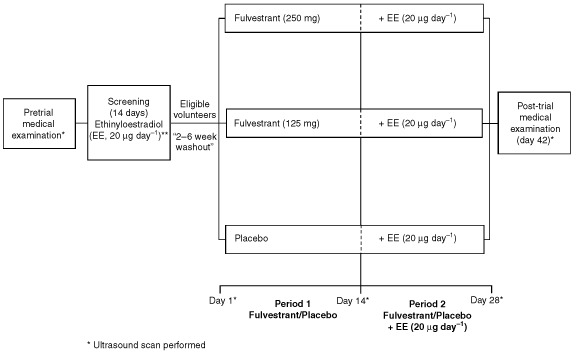
Study design. N.B. Fulvestrant administered as a single intramuscular injection, which provided continuous exposure over at least a 28-day period.

This was a double-blind, randomised, parallel-group, single-centre trial in healthy, postmenopausal volunteers (Figure 1

). The protocol was approved by the local ethics committee prior to volunteer recruitment. All volunteers provided signed informed consent before undergoing a routine pretrial medical examination, including ultrasound assessment to measure endometrial thickness.

Following the pretrial medical examination, volunteers were eligible to enter the screening period of the trial if they:

1. were aged between 45 and 60 years;
2. were postmenopausal (defined as not having experienced menstruation for more than 1 year and having serum luteinising hormone (LH), follicle-stimulating hormone (FSH) and oestradiol levels in the postmenopausal range);
3. had a normal clinical assessment including medical history and resting ECG;
4. had an endometrial thickness at baseline of ⩽4 mm, and had at least one ovary and a normal uterus;
5. weighed within 20% of their desired bodyweight;
6. had undergone a normal cervical smear within the last 5 years and had a normal mammogram within the last 3 years.

Individuals were excluded if they had a history of:

1. prior HRT;
2. oestrogen-dependent conditions including breast cancer;
3. disease affecting bone or steroid metabolism;
4. any conditions known to increase the risk of thromboembolic events;
5. use of drugs known to affect sex hormone status or steroid metabolism;
6. gastrointestinal, hepatic or renal disease that might interfere with absorption, metabolism and excretion of drugs.

### Screening

Eligible volunteers entered the screening period, during which time they received oral ethinyloestradiol 20 μg day^-1^ (two×10 μg tablets) at the same time each day for a period of 14 days. Only those volunteers with an endometrial response, defined as an endometrial thickness of ⩾8 mm after oestrogen treatment, were allowed to continue on the trial after a ‘washout period’ of 2–6 weeks following the first course of oestrogen. Volunteers were excluded from the randomised phase of the trial if their endometrial thickness did not return to baseline after the ‘washout period’.

### Randomisation

Eligible volunteers were randomised in a 1 : 1 : 1 ratio to receive fulvestrant 250 mg, fulvestrant 125 mg or matched placebo. Randomisation was in balanced blocks. The treatment given to each individual was determined by a random scheme prepared by the Biostatistics Group at AstraZeneca. All treatments were administered into the buttock as a single i.m. injection, which provided continuous exposure to fulvestrant over at least a 28-day period. Two weeks after administration of the randomised trial medication, all volunteers received a further 2-weeks exposure to ethinyloestradiol 20 μg day^−1^. Hence, the first 14-day exposure to fulvestrant (treatment period 1) assessed the effects of fulvestrant alone, while the subsequent 14-day period of exposure (treatment period 2) assessed the combination of fulvestrant 125 or 250 mg plus ethinyloestradiol, and ethinyloestradiol alone. No concomitant medication, other than simple analgesia, was allowed from 72 h before screening, day 1, until completion of the post-trial medical examination, without the prior consent of the investigator.

### Endometrial thickness

The primary endpoint of this trial was endometrial thickness. All volunteers underwent an ultrasound scan of the endometrium to determine endometrial thickness at the pretrial medical examination and after receiving 14 days of ethinyloestradiol (20 μg day^−1^) on day 14 of the screening period. Volunteers whose endometrium had not returned to baseline thickness after the screening period were not randomised. However, a further ultrasound could be performed after a longer ‘washout’ if the investigator considered it to be appropriate. Subsequent ultrasound scans were performed on days 14 (end of period 1), 28 (end of period 2) and 42 post-treatment with fulvestrant or placebo. The endometrial measurement, defined as the combined thickness of the two endometrial layers lying side by side, was performed through the thickest area of the endometrium. The measurement was performed in the longitudinal plane and then the transverse plane at the same location. The measurement markers were placed at the boundary between the myometrium and endometrium and the measurement recorded.

### Pharmacokinetic assessments

Venous blood was taken prior to dosing with either fulvestrant (125 mg or 250 mg) or placebo (day 1), 2 h after dosing (day 1) and on trial days 3, 7, 11, 14, 21 and 28. Determinations of maximum plasma concentration (C_max_), area under plasma concentration-time curve from zero to day 27 (AUC_(0–27)_) and time to maximum plasma concentration (t_max_) were made. The analyses were based on a validated high-performance liquid chromatography method with tandem mass spectrometry (Analytico Medinet BV, The Netherlands) (Data on file, AstraZeneca), the results of which have been presented previously (Robertson, 2000; Robertson *et al*, 2000).

### Safety

The safety assessments made in this trial were AEs, clinical chemistry, haematology and urinalysis. The AEs were recorded by the investigator and categorised according to COding Symbols for Thesaurus of Adverse Reaction Terms (COSTART) classification and body systems. An AE was defined as the development of any medical condition or deterioration of a pre-existing condition. The medical condition did not need to have had a causal relationship with exposure to the trial treatments and could be symptoms (e.g. nausea and chest pain) or abnormal results on investigation (e.g. blood tests, scans or ECG).

### Statistics

The size of the study was based upon the primary trial endpoint, endometrial thickness. Using data from previous trials, it was considered necessary for the trial to be able to detect a difference in endometrial thickness of 8.0 mm between the fulvestrant and placebo treated groups. Based upon an estimation of between-volunteer variability, it was calculated that nine volunteers per group would be required to allow an 85% chance of detecting an 8.0 mm difference in endometrial thickness between fulvestrant and placebo treated groups. Ten volunteers per group were therefore considered satisfactory to provide sufficient power to determine significant changes in endometrial thickness, while at the same time allowing for the possible withdrawal of one subject per group. As a result, a total of 30 volunteers were required.

Only the data obtained from the measurement of endometrial thickness were subject to formal statistical analysis (*t*-test). Non-compartmental pharmacokinetic analysis was performed on the plasma concentration-time data. C_max_ and t_max_ were determined from the fulvestrant plasma concentration-time profiles, and AUC_(0–27)_ was calculated using the linear trapezoidal rule. The data were presented using the following summary statistics: geometric mean (gmean), coefficient of variance (CV), gmean standard deviation (s.d.), arithmetic mean (calculated using untransformed data) and s.d. (calculated using untransformed data) depending on the parameter. t_max_ was presented as median, minimum, maximum.

## RESULTS

### Volunteer demographics

A total of 30 healthy, postmenopausal, female volunteers were recruited into the trial and received one course of 20 μg day^−1^ ethinyloestradiol for 14 days during screening. All 30 volunteers had a positive response to the administered oestrogen and were subsequently randomised as follows: 10 patients to receive fulvestrant 125 mg, 10 patients to receive fulvestrant 250 mg and 10 patients to receive placebo. The three treatment groups were well-matched for age, with the mean age (range) for the placebo, fulvestrant 125 mg and fulvestrant 250 mg treated patients being 56.8 years (48–64), 55.0 years (49–64) and 58.9 years (56–63), respectively. Similarly, mean weight and height were comparable between the three groups, with a mean weight (range) of 65.8 kg (54–76), 62.7 kg (48–76) and 61.1 kg (21–71), and a mean height (range) of 162.7 cm (153–169), 163.1 cm (153–171) and 161.1 cm (150–168), for the placebo, fulvestrant 125 mg and fulvestrant 250 mg treated patients, respectively.

There were no withdrawals or protocol violations during the trial. However, one patient was excluded from statistical analysis due to a protocol deviation. This patient took only one ethinyloestradiol tablet per day during the ethinyloestradiol treatment phase. Ten other volunteers had protocol deviations that did not lead to exclusion from statistical analysis. All 30 volunteers were included in the safety analyses.

### Endometrial thickness

At screening, all 30 volunteers had a normal postmenopausal baseline endometrial thickness of ⩽4 mm and responded to the screening oestrogen challenge with an increase in endometrial thickness to ⩾8 mm. Endometrial thickness after 14 days of 20 μg day^-1^ ethinyloestradiol varied between 8 and 12 mm except for one volunteer whose endometrial thickness had increased to 20 mm.

In all groups, the baseline value on day 1 was slightly elevated compared with the screening value, suggesting that the endometrial thickness after 14 days of oestrogen stimulation had not completely returned to preoestrogen levels, however the differences appeared to be within the level of variability observed for endometrial thickness at these time points. For all volunteers the endometrial thickness on day 1 of treatment was ⩽4 mm (Table 1

**Table 1:**
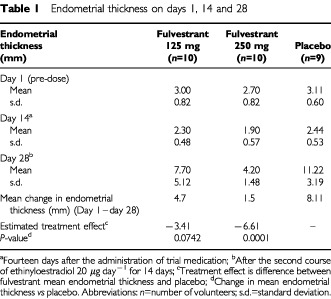
Endometrial thickness on days 1, 14 and 28

). Fourteen days after administration of i.m. fulvestrant, at a dose of either 125 mg or 250 mg, the mean endometrial thickness in each group was not clinically different from the screening value for that group, with no clinically significant differences between the groups (Table 1).

Following the 14-day treatment period 2, during which time ethinyloestradiol was administered in addition to fulvestrant (125 mg or 250 mg) and placebo, the mean endometrial thickness for the three groups was 7.70 mm for fulvestrant 125 mg plus ethinyloestradiol, 4.20 mm for fulvestrant 250 mg plus ethinyloestradiol, and 11.22 mm for placebo plus ethinyloestradiol, respectively (Table 1). There was a statistically significant difference between the fulvestrant 250 mg-treated group and the placebo-treated group for the change in mean endometrial thickness between day 1 and day 28 (*P*=0.0001) (Table 1). There was no statistically significant difference between the fulvestrant 125 mg group and the placebo group (*P*=0.0742) (Table 2

**Table 2:**
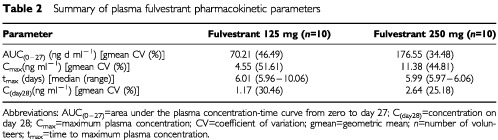
Summary of plasma fulvestrant pharmacokinetic parameters

). The mean endometrial thickness from pretrial to 42 days post-treatment with placebo or fulvestrant is plotted in Figure 2

**Figure 2:**
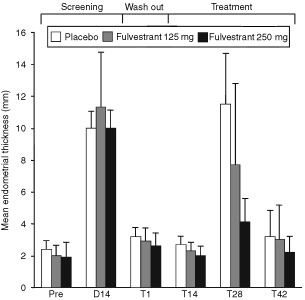
Mean endometrial thickness from premedical examination to trial day 42. Abbreviations: Pre=premedical examination; D14=after 14 days of ethinyloestradiol treatment; T1=treatment day 1 immediately before patients received either placebo or fulvestrant (125 mg or 250 mg); T14=day 14 when volunteers received their second challenge with ethinyloestradiol; T28=day 28 post-treatment at the end of the second ethinyloestradiol challenge; T42=day 42 post-treatment.

.

### Pharmacokinetics

**Figure 3:**
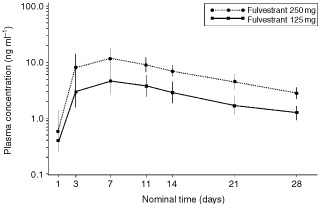
Geometric mean (s.d.) plasma concentrations of fulvestrant. Day 1 concentration was taken 2 h after dosing; day 14 concentration was taken pre-inflation of cuff.

A summary of the pharmacokinetic parameters is presented in Table 2. The gmean peak plasma concentrations of fulvestrant rose to 4.55 and 11.38 ng ml^-1^ approximately 6 days after i.m. injection of 125 mg and 250 mg fulvestrant, respectively (Figure 3

). By trial day 28, the plasma concentrations had declined approximately four-fold (Table 2). Exposure following the 250 mg dose of fulvestrant (AUC_0–27_) was approximately 2.5 times greater than that derived from the 125 mg dose. Higher plasma concentrations of fulvestrant (assessed on day 28) were associated with greater reductions in endometrial thickness.

### Safety

There were no serious AEs or events leading to volunteer withdrawal during this trial. Twenty-six volunteers reported a total of 77 AEs during the screening phase. During the first 14 days of the trial, 16 volunteers reported 28 AEs across all treatment groups, and during days 15 to 28, 22 volunteers reported 57 AEs across all treatment groups. Overall, the commonly reported AEs (i.e. those occurring in ⩾20% volunteers) were headache, leucorrhoea, breast pain and abdominal pain (Table 3

**Table 3:**
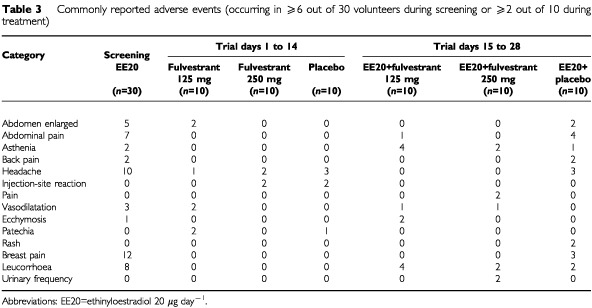
Commonly reported adverse events (occurring in ⩾6 out of 30 volunteers during screening or ⩾2 out of 10 during treatment)

).

The majority of AEs (105 out of a total of 162) occurred in volunteers receiving ethinyloestradiol alone either in the screening phase or in combination with placebo. The number of AEs reported during days 15 to 28 of treatment period 2, in the group receiving fulvestrant 250 mg plus ethinyloestradiol was less than half the number reported over the same time period in the group who received ethinyloestradiol plus placebo. Fulvestrant appeared to reduce the incidence of some of the ethinyloestradiol-induced AEs such as breast pain. Most of the AEs reported by volunteers receiving fulvestrant were considered to be treatment related and consisted of flushing, headache and injection-site reaction. There were no dose-related trends in the reporting of AEs.

## DISCUSSION

A reduction in oestrogenic stimuli forms the basis of treatment for many benign and malignant diseases of the breast and reproductive tract. This study was conducted to examine the effect of fulvestrant, a new ER antagonist, both alone and in combination with ethinyloestradiol, on the endometrium of healthy postmenopausal women.

Compared with volunteers who received unopposed ethinyloestradiol, those women who received the combination of fulvestrant and ethinyloestradiol had reduced endometrial thickening, demonstrating an antioestrogenic effect of fulvestrant on normal postmenopausal endometrium. The difference in endometrial thickening was clinically and statistically significant in volunteers who received the 250 mg dose of fulvestrant, the dose evaluated in the breast cancer setting. Although endometrial thickness was reduced in volunteers receiving fulvestrant 125 mg compared with the placebo group, the mean difference was smaller and failed to reach statistical significance, suggesting the response to fulvestrant was dose related.

Fulvestrant also demonstrated no oestrogen agonist effect on the endometrium during the short-term (14 day) period of administration. There were no clinically significant differences in endometrial thickness between the fulvestrant and placebo groups.

Overall, fulvestrant at both doses, either alone or in combination with ethinyloestradiol, was well tolerated. Most of the AEs were associated with ethinyloestradiol dosing or were considered not to be drug related. Fulvestrant appeared to reduce the number of ethinyloestradiol-related events providing further evidence for the antioestrogenic effects of this agent. The pharmacokinetic data were consistent with previous studies in patients with breast cancer (Howell *et al*, 1996; Robertson *et al*, 2000).

In conclusion, the results of this trial demonstrate an antioestrogenic effect of fulvestrant at doses of 125 and 250 mg on the endometrium. In addition, in the absence of oestrogen, fulvestrant demonstrated no oestrogen agonist effects during the 14-day period of administration. These data confirm that fulvestrant 250 mg, a dose known to be active in breast cancer therapy, is a well-tolerated oestrogen antagonist that is devoid of agonist activity on the endometrium in healthy, postmenopausal women.
